# Low sclerostin levels: a predictive marker of persistent inflammation in ankylosing spondylitis during anti-tumor necrosis factor therapy?

**DOI:** 10.1186/ar4055

**Published:** 2012-10-12

**Authors:** Carla GS Saad, Ana CM Ribeiro, Julio CB Moraes, Liliam Takayama, Celio R Goncalves, Marcelo B Rodrigues, Ricardo M de Oliveira, Clovis A Silva, Eloisa Bonfa, Rosa MR Pereira

**Affiliations:** 1 Division of Rheumatology, Faculdade de Medicina da Universidade de São Paulo, Av Dr Arnaldo, 455 3° andar sala 3105, São Paulo, Brazil; 2 Department of Radiology, Faculdade de Medicina da Universidade de São Paulo, Av Dr Enéas de Carvalho Aguiar 250, São Paulo, Brazil; 3 RDO Diagnósticos Médicos, Av Brasil 1150, São Paulo, Brazil

## Abstract

**Introduction:**

Sclerostin levels have been reported to be low in ankylosing spondylitis (AS), but there is no data regarding the possible role of this Wnt inhibitor during anti-tumor necrosis factor (TNF) therapy. The present study longitudinally evaluated sclerostin levels, inflammatory markers and bone mineral density (BMD) in AS patients under anti-TNF therapy.

**Methods:**

Thirty active AS patients were assessed at baseline, 6 and 12 months after anti-TNF therapy regarding clinical parameters, inflammatory markers, BMD and baseline radiographic damage (mSASSS). Thirty age- and sex-matched healthy individuals comprised the control group. Patients' sclerostin levels, sclerostin binding low-density lipoprotein receptor-related protein 6 (LRP6) and BMD were evaluated at the same time points and compared to controls.

**Results:**

At baseline, AS patients had lower sclerostin levels (60.5 ± 32.7 vs. 96.7 ± 52.9 pmol/L, *P *= 0.002) and comparable sclerostin binding to LRP6 (*P *= 0.387) than controls. Improvement of Bath Ankylosing Spondylitis Disease Activity Index (BASDAI), Bath Ankylosing Spondylitis Functional Index (BASFI), Bath Ankylosing Spondylitis Metrology Index (BASMI), Ankylosing Spondylitis quality of life (ASQoL) was observed at baseline vs. 6 vs. 12 months (*P *< 0.01). Concomitantly, a gradual increase in spine BMD (*P *< 0.001) and a positive correlation between baseline mSASSS and spine BMD was found (r = 0.468, *P *< 0.01). Inflammatory parameters reduction was observed comparing baseline vs. 6 vs. 12 months (*P <*0.01). Sclerostin levels progressively increased [baseline (60.5 ± 32.7) vs. 6 months (67.1 ± 31.9) vs. 12 months (72.7 ± 32.3) pmol/L, *P <*0.001]. At 12 months, the sclerostin levels remained significantly lower in patients compared to controls (72.7 ± 32.3 vs. 96.70 ± 52.85 pmol/L, *P *= 0.038). Moreover, sclerostin serum levels at 12 months were lower in the 10 patients with high C reactive protein (CRP) (≥ 5 mg/l) compared to the other 20 patients with normal CRP (*P *= 0.004). Of note, these 10 patients with persistent inflammation also had lower sclerostin serum levels at baseline compared to the other patients (*P *= 0.023). Univariate logistic regression analysis demonstrated that AS patients with lower sclerostin serum levels had an increased risk to have high CRP at 12 months (odds ratio = 7.43, 95% CI 1.23 to 45.01, *P *= 0.020) than those with higher sclerostin values.

**Conclusions:**

Persistent low sclerostin levels may underlie continuous inflammation in AS patients under anti-TNF therapy.

## Introduction

Ankylosing spondylitis (AS) is a chronic inflammatory rheumatic disease characterized by new bone growth that leads to syndesmophyte formation and subsequent vertebral ankylosis [1].

Several biomarkers, including inflammatory mediators and factors that reflect joint tissue turnover, have been evaluated in order to determine their relevance in AS clinical prognosis and therapy response [2]. In this disease, the search for biomarkers that regulate bone formation is essential to understand the underlying mechanism regulating bone growth and the inflammatory process. Bone formation in AS has recently been associated with upregulation of wingless (Wnt) signaling, a pathway implicated in osteoblastogenesis [3]. Natural inhibitors of Wnt, such as sclerostin and Dkk-1, neutralize Wnt pathway activity and are critically important in normal bone homeostasis, particularly osteoblastic new bone formation [3-8]. Interestingly, TNFα, an inflammatory mediator, is overexpressed in sacroiliac joints [9] and may also regulate the Wnt/β catenin signaling pathway and the Wnt proteins [10,11].

Anti-TNFα agents are efficient therapeutic options for treating axial disease and peripheral arthritis in spondyloarthritis [12-16]. However, TNF-blockers do not appear to inhibit structural damage, bone formation or radiographic progression in AS patients [17-21], and a role for inflammation particularly at sites that develop bony proliferation has been suggested [5,21].

In addition, the recent observation that sclerostin is nearly absent in AS osteocytes [5] supports the hypothesis that natural inhibitors of the Wnt pathway are relevant players in the pathogenesis of AS [3,5-8]. However, there are no data on the relevance of this Wnt inhibitor in anti-TNF therapy response and inflammation. In this regard, combinations of biomarkers reflecting an inflammatory and reparative process is critical to clarify the scope of anti-TNFα therapeutic benefit. We therefore assessed sclerostin serum levels, clinical parameters, inflammatory markers and bone mineral density (BMD) in active AS patients during anti-TNF therapy.

## Materials and methods

### Subjects

Thirty consecutive active AS patients followed at the Spondyloarthritis Outpatient Clinic and referred to the Infusion Center were evaluated at baseline, 6 and 12 months after the initiation of anti-TNF therapy at the standard dose; nineteen patients were taking infliximab, nine adalimumab, and two etanercept. All of them fulfilled the modified New York classification criteria for definite AS [22]. Thirty sex- and age-matched healthy individuals comprised the control group.

Inclusion criteria for patients were presence of active disease with a Bath AS Disease Activity Index (BASDAI) ≥ 4 and/or high inflammatory parameters refractory to nonsteroidal anti-inflammatory drugs (NSAIDs) and disease-modifying antirheumatic drugs, and referral for anti-TNF therapy. Exclusion criteria for both groups were other diseases that may have an effect on bone metabolism such as hyperthyroidism, malabsorption syndrome, kidney and liver failure, alcohol, prior or current use of bisphosphonates and current glucocorticoid use (> 5 mg/day) for patients and glucocorticoid use for control group. Baseline AS treatment with NSAIDs and/or disease-modifying antirheumatic drugs (sulfasalazine/methotrexate) was stable for at least 3 months and remained stable throughout the study.

### Study design

Longitudinal sclerostin serum levels/binding, BMD, inflammatory markers and clinical outcomes were evaluated at baseline, 6 and 12 months after treatment. Radiographic analysis in AS patients was assessed at baseline. BMD and sclerostin measurements were also analyzed at baseline and 12 months in the controls.

This study was approved by the Local Ethics Committee on Human Research at the University of São Paulo. All participants gave written informed consent in accordance with the Declaration of Helsinki.

### Clinical Outcomes of AS

Clinical indices including BASDAI, Bath AS Functional Index (BASFI), Bath AS Metrology Index (BASMI) and AS quality of life questionnaire (ASQoL) were measured at baseline and at each follow-up visit, as recommended previously [23].

### Laboratory assessment of inflammatory parameters

Inflammation was evaluated by erythrocyte sedimentation rate (ESR) in mm/h and C-reactive protein (CRP) in mg/L. Serum matrix metalloproteinase 3 (MMP-3) levels were measured in ng/mL using a commercially available ELISA (human total MMP-3 immunoassay ELISA kit, R&D Systems, Minneapolis, MN, USA), according to the manufacturer's recommendations.

### Laboratory assessment of hormones

Hormonal determinations were performed at study entry in AS patients and controls. Serum follicle stimulating hormone (FSH) was analyzed in all patients and controls, total testosterone in men and estradiol in women. All assays were performed using the Cobas e 411 Immunoassay Analyzer, Roche Diagnostics GmbH (Mannheim, Germany), according to the manufacturer's instructions. The intra- and inter-assay coefficients of variation (CVs) were less than 6.0%.

### Laboratory evaluation of the Wnt signaling pathway

Serum samples were obtained from all patients and controls after fasting and stored at -80°C until further analysis. Serum sclerostin levels were measured by a commercial sandwich ELISA KIT (Lot Y105, reference control: 90.5 (84 ± 25) pmol/L, Biomedica, Vienna, Austria) [24]. The intra- and inter-assay CV was 5% and 6%, respectively. Binding of sclerostin to the low-density lipoprotein receptor-related protein 6 (LRP6) in AS patients and controls was measured using a functional ELISA at baseline and 12 months. Briefly, microtiter plates were coated with 5 μg/mL of a chimeric human LRP6-Fc (recombinant human LRP6-Fc chimera; R&D Systems) prior to the addition of serum samples, and detection was performed using human anti-sclerostin biotinylated antibody (Biomedica). The results were expressed in optical density (OD_450_). The intra- and inter-assay CV for this analysis was 5% and 3 to 6%, respectively.

### Radiological assessment

Radiographic images of the cervical and lumbar spine were evaluated in AS patients concomitantly by two trained readers (a rheumatologist and a musculoskeletal radiologist) with agreement, using the modified Stoke Ankylosing Spondylitis Spine Score (mSASSS) [25] at baseline.

### Bone densitometry

BMD was measured in patients and healthy controls by dual-energy X-ray absorptiometry (DXA) using Hologic densitometry equipment (QDR 4500 densitometer) (Hologic Inc., Bedford, MA, USA) at the lumbar spine, femoral neck, and total femur, at the Bone Metabolism Laboratory of the Rheumatology Division. All BMD measurements were performed by the same experienced technologist. Precision error for BMD measurements was determined based on standard International Society for Clinical Densitometry (ISCD) protocols [26]. We calculated the least significant change with 95% confidence to be 0.033 g/cm^2 ^at the spine, 0.047 g/cm^2 ^at the femoral neck, and 0.039 g/cm^2 ^at the total femur. BMD was expressed in g/cm^2^.

### Statistical analysis

Results are presented as mean and SD for continuous variables and percentages for categorical variables. Data for continuous variables were compared by the *t*-test to evaluate differences between patients and controls, since all parameters had a normal distribution. Clinical, laboratory and densitometric data were compared at baseline, 6 and 12 months using analysis of variance (ANOVA) with the Huynh-Feldt correction when it was applicable, and the Bonferroni or Frideman tests for multiple comparisons. The Kruskal-Wallis test was applied to compare sclerostin serum levels at 12 months among patients with normal CRP values (< 5 mg/L), high CRP values (≥ 5 mg/L) and the control group. Univariate logistic regression analysis was performed to evaluate the influence of sclerostin serum levels in CRP at 12 months. Differences between categorical variables were evaluated using the chi-squared or Fisher exact test. Spearman's analysis was used for correlation analysis. Statistical significance was established at *P *< 0.05.

## Results

### Baseline demographics, disease characteristics and BMD in patients and controls

At baseline, AS patients and the control group had a mean age ± SD of 35.7 ± 11.0 years; 80% were men and 86.6% were Caucasian. Patients and healthy controls had comparable weight (72.6 ± 13.6 vs. 75.8 ± 10.3 Kg, *P *= 0.313), height (1.68 ± 0.09 vs. 1.71 ± 0.06 cm, *P *= 0.150) and body mass index (BMI) (25.7 ± 4.6 vs. 25.8 ± 2.9 Kg/m^2^, *P *= 0.909). The mean disease duration was 11.9 ± 9.3 years; one-third (33.3%) of patients had only axial involvement and 66.6% of patients had axial and peripheral involvement with active synovitis at study entry. Baseline clinical assessment revealed high disease activity (BASDAI 5.11 ± 2.1, BASFI 5.4 ± 2.7, BASMI 4.06 ± 2.7) and ASQoL score of 10.03 ± 5.2. AS patients and controls were comparable regarding serum FSH (6.81 ± 7.79 vs. 4.80 ± 2.81 IU/L in men, *P *= 0.241, and 23.32 ± 29.72 vs. 26.3 ± 48.43 IU/L in women, *P *= 0.900), testosterone (6.86 ± 2.57 vs. 6.72 ± 2.54 ng/mL, *P *= 0.841), estradiol (112.74 ± 34.15 vs. 145.96 ± 115.63 pg/mL, *P *= 0.515). Patients and healthy subjects had comparable BMD at the lumbar spine (1.032 ± 0.175 vs. 1.082 ± 0.111 g/cm^2^, *P *= 0.201), femoral neck (0.864 ± 0.161 vs. 0.888 ± 0.135 g/cm^2^, *P *= 0.547) and total femur (0.991 ± 0.165 vs. 1.052 ± 0.111 g/cm^2^, *P *= 0.099).

### Sclerostin serum levels and binding in patients and controls

At baseline, AS patients had significantly lower sclerostin levels (60.5 ± 32.7 vs. 96.7 ± 52.9 pmol/L, *P *= 0.002) compared to healthy controls, and similar binding of sclerostin to LRP6 (0.61 ± 0.13 vs. 0.66 ± 0.27 OD_450_, *P *= 0.387).

Also at baseline, no difference was observed in sclerostin levels between patients with peripheral vs. non-peripheral involvement (69.9 ± 29.6 vs. 55.7 ± 33.9 pmol/L, *P *= 0.269), and patients with > 5 years versus < 5 years of disease duration (48.13 ± 33.50 vs. 65.76 ± 31.71 pmol/L, *P *= 0.180).

### Correlation between baseline radiographic damage (mSASSS) and lumbar spine BMD

**Figure 1:**
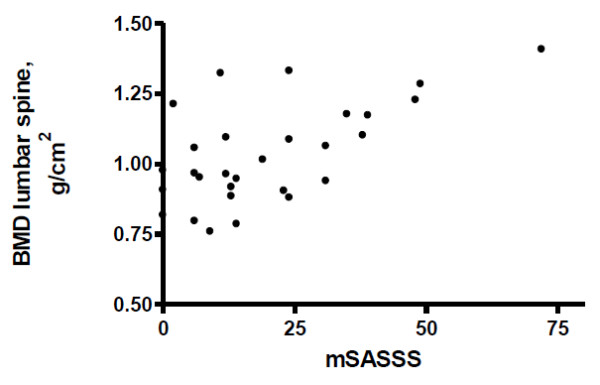
**Spearman's correlation between modified Stoke ankylosing spondylitis spinal score (mSASSS) and lumbar spine bone mineral density (BMD)**.

Radiographic analyses revealed mean mSASSS of 21.1 ± 17.8 units at baseline. mSASSS at baseline correlated with BMD of the lumbar spine at baseline (r = 0.468, *P *< 0.01) (Figure 1).

### Longitudinal improvement of clinical parameters and reduction of inflammatory parameters after anti-TNF treatment

A significant clinical improvement was observed comparing baseline vs. 6 months vs. 12 months in the following clinical variables: BASDAI, BASFI, BASMI and AsQoL (*P *< 0.01) (Table 1).

**Table 1:** Changes in clinical assessments, bone mineral density and laboratory analysis following anti-TNF treatment in AS patients

	Baseline	6 months	12 months	*P*
BASDAI	5.11 ± 2.1	2.78 ± 2.4	2.78 ± 2.5	< 0.001
BASFI	5.40 ± 2.7	2.96 ± 2.6	2.85 ± 2.7	< 0.001
BASMI	4.06 ± 2.7	3.23 ± 2.6	3.07 ± 2.7	0.003
AsQoL, score	10.03 ± 5.2	6.43 ± 6.4	6.40 ± 6.05	0.001
ESR, mm/hr	23.75 ± 23.1	8.16 ± 15.6	7.3 ± 13.1	< 0.001
C-reactive protein, mg/dL	37.03 ± 47.9	3.35 ± 4.1	7.21 ± 20.1	< 0.001
MMP-3, ng/mL	48.6 ± 42.5	21.6 ± 22.5	23.3 ± 30.8	< 0.001
BMD lumbar spine, g/cm^2^	1.032 ± 0.175	1.049 ± 0.169	1.065 ± 0.169	< 0.001
BMD femoral neck, g/cm^2^	0.864 ± 0.160	0.865 ± 0.159	0.864 ± 0.161	0.051
BMD total femur, g/cm^2^	0.990 ± 0.165	0.986 ± 0.165	0.999 ± 0.171	0.981
Sclerostin, pmol/L	60.5 ± 32.7	67.1 ± 31.9	72.7 ± 32.3	< 0.001
Sclerostin binding to LRP6, OD_450_	0.61 ± 0.13	ND	0.60 ± 0.14	0.472

Values are expressed as mean (SD). AS, ankylosing spondylitis; BASDAI, Bath AS Disease Activity Index; BASFI, Bath AS Functional Index; BASMI, Bath AS Metrology Index; ASQoL, AS quality of life questionnaire; BMD, bone mineral density; ESR, erythrocyte sedimentation rate; MMP-3, matrix metalloproteinase 3; LRP6, low-density lipoprotein receptor-related protein 6; OD, optical density; ND, not done.

A concomitant reduction in ESR, CRP and MMP-3 levels (*P *< 0.001) was observed after anti-TNF therapy comparing baseline vs. 6 months vs. 12 months (Table 1). No difference in these inflammatory parameters was found comparing 6 and 12 months (*P *> 0.05). At 12 months 10 (33.3%) patients continued to have high CRP (≥ 5 mg/L).

### Longitudinal increase in spine BMD 12 months after anti-TNF treatment in AS patients

A significant increase in lumbar spine BMD (*P *< 0.001) was found from baseline vs. 6 months vs. 12 months after anti-TNF therapy, whereas no significant change was observed for femoral neck and total femur BMD (Table 1).

In the control group, no difference was observed in lumbar spine BMD comparing baseline and 12 months (1.082 ± 0.111 vs. 1.079 ± 0.117 g/cm^2^, *P *= 0.681).

### Longitudinal evaluation of sclerostin serum levels and binding after anti-TNF treatment

**Figure 2:**
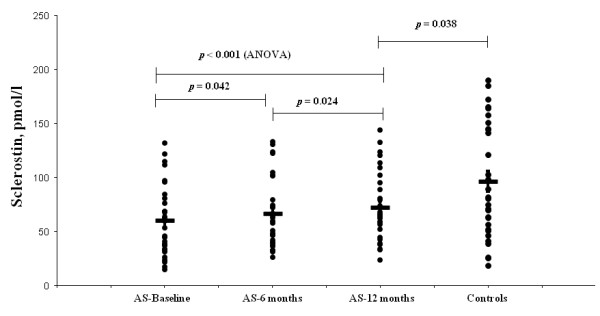
**Comparisons of sclerostin serum levels in ankylosing spondylitis (AS) patients at baseline, 6 months and 12 months after anti-TNF therapy and in healthy controls**.

Serum levels of sclerostin gradually increased from baseline vs. 6 months vs. 12 months after biological treatment (60.5 ± 32.7 vs. 67.1 ± 31.9 vs. 72.7 ± 32.3 pmol/L, *P <*0.001) (Table 1 and Figure 2). These levels continued to increase between 6 and 12 months of anti-TNF therapy (*P *= 0.024). However, at 12 months, sclerostin levels remained significantly lower in patients compared to controls (72.7 ± 32.3 vs. 96.70 ± 52.9 pmol/L, *P *= 0.038) (Figure 2). No change was observed in binding of sclerostin to LRP6 at baseline and after 12 months of anti-TNF treatment (0.61 ± 0.13 vs. 0.60 ± 0.14 OD_450_, *P *= 0.472).

In the control group no difference was detected in sclerostin serum levels comparing baseline and 12 months (96.7 ± 52.9 vs. 99.6 ± 63.5 pmol/L, *P *= 0.38).

### Sclerostin levels in patients with and without persistent high CRP at 12 months

**Figure 3:**
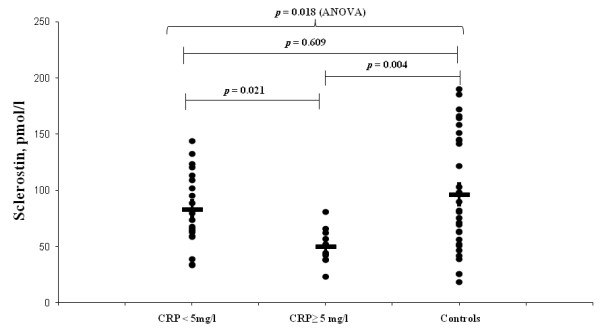
**Comparisons of sclerostin serum levels in ankylosing spondylitis (AS) patients with C-reactive protein (CRP) < 5 mg/dL and CRP ≥ 5 mg/dL 12 months after anti-TNF therapy and in healthy controls**.

Further analysis was performed comparing patients with persistent inflammation (CRP ≥ 5 mg/L) and those without inflammation (CRP < 5 mg/L) at 12 months. Sclerostin serum levels at 12 months were lower in the 10 patients with high CRP compared to the other 20 patients with normal levels (50.8 ± 16.6 vs. 83.6 ± 33.0 pmol/L, *P *= 0.021). Reinforcing this finding, a negative Spearman correlation between sclerostin and CRP (r = -0.57, *P *= 0.001) and ESR (r = -0.59, *P *< 0.001) was observed. Moreover, at 12 months, patients with normal CRP had comparable values of sclerostin levels to the control group (83.6 ± 33.0 vs. 96.7 ± 52.9 pmol/L, *P *= 0.609) while patients with persistent high CRP had significantly lower sclerostin levels than the control group (50.8 ± 16.6 vs. 96.7 ± 52.9 pmol/L, *P *= 0.004) (Figure 3).

### Sclerostin levels at baseline as predictor of persistent inflammation

The association between baseline predictors of anti-TNF response (age, disease duration, BASDAI, mSASSS, spine BMD, CRP level, ESR level, MMP-3 and sclerostin) and persistent inflammation at 12 months (10 patients with CRP ≥ 5 mg/L vs. 20 patients with CRP < 5 mg/L) on univariate analysis revealed that only baseline low sclerostin levels were significantly associated with persistent inflammation at 12 months (41.4 ± 19.5 vs. 70.0 ± 33.3 pmol/L, *P *= 0.023) (Table 2). Univariate logistic regression analysis demonstrated that AS patients with lower sclerostin serum levels (< 65 pg/mL, AS median time-averaged sclerostin serum levels) had an increased risk of high CRP at 12 months (odds ratio 7.43, 95% CI 1.23 to 45.01, *P *= 0.020) than those with higher sclerostin values.

**Table 2:** AS baseline parameters considered as predictors of anti-TNF response and persistent inflammation (CRP ≥ 5 mg/L) at 12 months

Baseline parameters	CRP ≥ 5 mg/L *n *= 10	CRP < 5 mg/L *n *= 20	*P*
Age, years	31.6 ± 10.8	37.7 ± 10.8	0.157
Disease duration, years	10.6 ± 8.9	12.5 ± 9.4	0.598
mSASSS	16.5 ± 14.9	23.35 ± 19.0	0.329
BMD lumbar spine, g/cm^2^	1.020 ± 0.191	1.040 ± 0.168	0.771
BASDAI	4.89 ± 2.1	5.56 ± 2.2	0.415
C-reactive protein, mg/L	58.13 ± 71.1	26.5 ± 27.4	0.166
ESR, mm/hr	38.5 ± 34.9	16.7 ± 9.8	0.176
MMP-3, ng/mL	70.2 ± 52.3	37.8 ± 33.0	0.090
Sclerostin, pmol/L	41.4 ± 19.5	70.0 ± 33.3	0.023

Values are expressed as mean (SD). AS, ankylosing spondylitis; mSASSS, modified Stoke Ankylosing Spondylitis Spine Score; BMD, bone mineral density; BASDAI, Bath AS Disease Activity Index; ESR, erythrocyte sedimentation rate; MMP-3, matrix metalloproteinase 3.

## Discussion

This is the first study to identify low sclerostin serum levels as a possible marker of persistent inflammation in AS patients under anti-TNF therapy. An important advantage of the present study design was the exclusion of diseases and drugs that may interfere in bone metabolism since kidney failure, hyperparathyroidism [27] and bisphosphonates [28] could regulate sclerostin expression in osteocytes and consequently affect the sclerostin serum levels. Also, the normal hormonal status of AS patients excludes the interference of this confounding variable in our results since FSH serum levels were recently identified as a stronger determinant of sclerostin serum levels [29].

Sclerostin is expressed almost exclusively in bone [30,31], specifically by osteocytes, and it is a key inhibitor of osteoblast activity by binding LRP5/LRP6 and inhibiting canonical Wnt signaling [4,32]. This Wnt signaling increases bone formation by stimulating osteoblast proliferation and differentiation [4]. We confirmed and extended the previous observation of a single study that serum sclerostin is lower in AS patients compared to healthy controls [5].

In addition, we demonstrated that TNF blockage induces a gradual increase in the level of this inhibitor but these levels at 12 months remained lower than healthy individuals. This finding suggests that sclerostin may underlie the persistent syndesmophyte bone formation reported for this disease, despite anti-TNF therapy [17-21]. This hypothesis is reinforced by the finding of a gradual increase in spine BMD which was correlated with baseline mSASSS. Further longitudinal studies are necessary to determine if sclerostin normalization is associated with reduced long-term radiographic progression.

Cross-talk between TNF and sclerostin appears to occur, therefore the regulation of this Wnt inhibitor factor could be influenced by inflammation and/or resolution of this process in AS patients [33]. The persistent low sclerostin serum levels restricted to the subgroup of patients with continuous high inflammatory parameters suggest that osteocyte dysfunction associated with AS disease [5] was not reverted in these patients in spite of TNF blockage. Moreover, baseline sclerostin levels seemed to be predictive of anti-TNF inflammatory response, since AS patients with higher baseline sclerostin levels had a rapid inflammatory response as expected for this treatment [12-16] and a concomitantly gradual increase in this Wnt inhibitor.

We speculated therefore that the mechanism involved in the syndesmophyte development is probably associated with persistent inflammation localized in the vertebral corner (cortical bone) with low sclerostin levels and a subsequent increase of bone formation at this site [34]. In contrast, the bone loss in the vertebra body previously reported in this disease, and not observed in the present study, could be related to osteoclast activation, mainly due to receptor activator of the nuclear factor kappa-B ligand (RANKL) pathway, possibly as a result of inflammation in the trabecular bone, or due to the known reduced mobility and physical activity of these patients [34,35].

The binding of sclerostin to LPR6 was comparable to controls and was not influenced by anti-TNF treatment, suggesting that the observed low level in patients is not due to hampered receptor binding. In contrast, concerning Dkk-1, another molecule that controls osteoblastogenesis, the defect of this inhibitor in AS patients seems to be associated with the reduced affinity to LPR6 [3,6,8].

## Conclusions

In conclusion, our results demonstrated that persistent low sclerostin serum levels are associated with continuous inflammation in AS patients under anti-TNF therapy. Further studies are necessary to determine if this alteration may be a marker of inflammation underlying bone formation in AS patients during anti-TNF therapy.

## Abbreviations

ANOVA: analysis of variance; AS: ankylosing spondylitis; ASQoL: Ankylosing Spondylitis quality of life questionnaire; BASDAI: Bath Ankylosing Spondylitis Disease Activity Index; BASFI: Bath Ankylosing Spondylitis Functional Index; BASMI: Bath Ankylosing Spondylitis Metrology Index; BMD: bone mineral density; CV: coefficient of variation;CRP: C-reactive protein; DXA: dual-energy X-ray absorptiometry; ELISA: enzyme-linked immunosorbent assay; ESR: erythrocyte sedimentation rate; FSH: serum follicle stimulating hormone; ISCD: International Society for Clinical Densitometry; LRP6: low-density lipoprotein receptor-related protein 6; MMP-3: matrix metalloproteinase 3; mSASSS: modified Stoke Ankylosing Spondylitis Spine Score; NSAIDs: non-steroidal anti-inflammatory drugs; OD_450_: optical density; TNF: tumor necrosis factor; Wnt: wingless.

## Competing interests

The authors declare that they have no competing interests.

## Authors' contributions

CGSS: study concept and design, assistance in subject recruitment, acquisition of subjects and data, interpretation of data and drafting the manuscript. ACMR: assistance in subject recruitment, acquisition of data. JCBM: assistance in subject recruitment, acquisition of data. LT: laboratory and densitometry data. CRG: assistance in subject recruitment. MBR: radiographic analysis. RMO: laboratory data. CAS: study concept and design. EB: study concept and design, interpretation of data and revision of the manuscript. RMRP: study concept and design, acquisition of subjects and data, interpretation of data and drafting the manuscript. All authors have read the latest version of the manuscript and gave final approval to this version.
